# Encapsulation of an anticancer drug Isatin inside a host nano-vehicle SWCNT: a molecular dynamics simulation

**DOI:** 10.1038/s41598-021-98222-2

**Published:** 2021-09-21

**Authors:** Maryam Zarghami Dehaghani, Farrokh Yousefi, Farzad Seidi, Babak Bagheri, Amin Hamed Mashhadzadeh, Ghasem Naderi, Amin Esmaeili, Otman Abida, Sajjad Habibzadeh, Mohammad Reza Saeb, Maksym Rybachuk

**Affiliations:** 1 Jiangsu Co-Innovation Center of Efficient Processing and Utilization of Forest Resources and Joint International Research Lab of Lignocellulosic Functional Materials, Nanjing Forestry University, Nanjing, 210037 China; 2 Department of Physics, University of Zanjan, 45195-313 Zanjan, Iran; 3 Department of Chemical and Biomolecular Engineering, Korea Advanced Institute of Science and Technology (KAIST), Daejeon, 305-701 Republic of Korea; 4 Center of Excellence in Electrochemistry, School of Chemistry, College of Science, University of Tehran, Tehran, Iran; 5 Iran Polymer and Petrochemical Institute (IPPI), Tehran, Iran; 6 Department of Chemical Engineering, College of the North Atlantic—Qatar, 24449 Arab League St, P.O. Box 24449, Doha, Qatar; 7 College of Engineering and Technology, American University of the Middle East, Egaila, Kuwait; 8 Department of Chemical Engineering, Amirkabir University of Technology (Tehran Polytechnic), Tehran, Iran; 9 Department of Chemical Engineering, McGill University, 3610 University Street, Montreal, QC H3A 0C5 Canada; 10 School of Engineering and Built Environment, Griffith University, 170 Kessels Rd, Nathan, QLD 4111 Australia; 11 Queensland Micro- and Nanotechnology Centre, Griffith University, West Creek Road, Nathan, QLD 4111 Australia

**Keywords:** Targeted therapies, Carbon nanotubes and fullerenes, Computational science

## Abstract

The use of carbon nanotubes as anticancer drug delivery cargo systems is a promising modality as they are able to perforate cellular membranes and transport the carried therapeutic molecules into the cellular components. Our work describes the encapsulation process of a common anticancer drug, Isatin (1H-indole-2,3-dione) as a guest molecule, in a capped single-walled carbon nanotube (SWCNT) host with chirality of (10,10). The encapsulation process was modelled, considering an aqueous solution, by a molecular dynamics (MD) simulation under a canonical NVT ensemble. The interactions between the atoms of Isatin were obtained from the DREIDING force filed. The storage capacity of the capped SWCNT host was evaluated to quantify its capacity to host multiple Isatin molecules. Our results show that the Isatin can be readily trapped inside the volume cavity of the capped SWCNT and it remained stable, as featured by a reduction in the van der Waals forces between Isatin guest and the SWCNT host (at approximately − 30 kcal mol^−1^) at the end of the MD simulation (15 ns). Moreover, the free energy of encapsulation was found to be − 34 kcal mol^−1^ suggesting that the Isatin insertion procedure into the SWCNT occurred spontaneously. As calculated, a capped SWCNT (10,10) with a length of 30 Å, was able to host eleven (11) molecules of Isatin, that all remained steadily encapsulated inside the SWCNT volume cavity, showing a potential for the use of carbon nanotubes as drug delivery cargo systems.

## Introduction

Cancer is the second-leading cause of death in the world^1^ and several therapeutic approaches have been developed to obviate the cancer cells from a body, including the use of surgical, radiation therapy and chemotherapy methods^2,3^. Despite the widespread application of chemotherapy in cancer treatment, the drawbacks of using anticancer drugs including their toxic side effects on fast-growing healthy cells, and multi-drug resistance are believed to limit the application of chemotherapy in cancer treatment^4,5^. Nanoscale delivery systems are promising modalities that have a potential to overcome the barriers associated with chemotherapy treatment by delaying drug resistance^6,7^, improving drug release profile into tumor micro-environment^8–11^, facilitating penetration of drugs into cancer cells and tissues^12^, while shielding drugs from premature chemical reactions and/or an induced physical tension^13,14^ and, most importantly, are able to apportion the required dosage of the drugs via their unique carrier profile^15,16^.

An open-ended or end-capped, carbon nanotubes (CNTs) have attracted a significant attention as possible drug nano-carries owing to their remarkable features including mechanical robustness and exceptionally large surface area per unit mass ratios, large enough to provide an ample volume for possible encapsulation and adsorption of nano-sized media such as drug molecules, compact storage and delivery characteristics^17^, and a relatively uncomplicated functionalization profile that allows CNTs to be doped or grafted with desired chemical structures and/or molecules^18–20^. Single-walled CNTs (SWCNTs) are *sp*^2^ hybridized allotropes of carbon, similar to fullerenes^21^. The structure of a SWCNT is comprised of 6-membered carbon rings, as in graphite^22^.The cylindrical SWCNTs may have one or both ends capped with a hemisphere of the buckyball or fullerene structure^23^.

A number of theoretical studies were performed recently to evaluate the performance of SWCNTs as possible host nano-vehicles for anticancer drug delivery including the study by Moradnia et al*.*^24^ that evaluated the adsorption function of Gemcitabine (AKA. Gemzar) anticancer drug on the surface of the functionalized SWCNT. The study evaluated the van der Waals (vdW), electrostatic (Elec), and binding free energy energies for the interaction of Gemcitabine employing a molecular dynamics (MD) simulation. The negative value for the free energy and vdW energy confirmed the conformational stability of Gemcitabine on the surface of SWCNTs. Roosta et al^25^ evaluated the changes in the Elec and vdW energies during the encapsulation process of the Cisplatin inside the CNT by using MD simulation. They reported that the vdW contribution to solute–solvent interaction energy and Elec contribution to solute–solvent interaction energy were equal to the values of − 0.322 kcal mol^−1^ and − 0.260 kcal mol^−1^, respectively. Maleki et al*.*^26^, studied the conformational attachment of another an anticancer drug Doxorubicin (AKA. Adriamycin, Rubex) on a SWCNT. Their calculations for vdW and Elec energies between the host SWCNT and Doxorubicin guest have shown that the value of the Elec energy was zero while the vdW interaction was solely responsible for the adsorption process. Mirsalari et al*.*^27^ studied the adsorption of the anticancer drug Dacarbazine into the CNT by means of MD simulation. They reported that Decarbazine was inserted spontaneously inside the cavity of the CNT so that the vdW energy decreased and reached the value of − 160 kJ mol^−1^ at the end of the simulation. Also, the adsorption of other anticancer drugs including Sunitinib (AKA. Sutent, SU11248), Streptozotocin or Streptozocin (INN, USP) (STZ), and Sorafenib (AKA. Nexavar) on functionalized SWCNTs were studied using the MD simulation by Dehneshin et al*.*^28^. The adsorption of Sorafenib on SWCNTs occurred through the π–π* stacking and hydrogen (H-) bonding, whereas STZ was absorbed exclusively through H-bonding. The interactions between anticancer drug Penicillamine (AKA. Cuprimine) and SWCNT was investigated using the MD simulation by Shaki et al*.*^11^; their study have shown that the interaction energies had negative values confirmed that the adsorption process of Penicillamine guest on SWCNT was exothermic and spontaneous. The co-adsorption process of Doxorubicin with various dye molecules on/into the SWCNT (30,0) is studied by using MD simulation by Panczyk et al*.*^29^. They reported that the drug can be released in acidic environment due to the presence of dye molecules facilitating the free energy barrier elimination against release. In another works done by the same team^30^, it was predicted that the Cisplatin can be adsorbed on SWCNT with free energy of − 25 kJ mol^−1^. Wolski et al*.*^31^ investigated the encapsulation of Carmustine anticancer drug inside the SWCNT, then capped it using functionalized magnetic nanoparticles by means of MD simulation. It was observed that the magnetic nanoparticle having diameters larger than 35 Å were able to absorb huge amounts of energy from the magnetic field and cause the release of the drug.

Considering the main concepts understood from the recent theoretical studies^11,26,28^ on the use of SWCNT-anticancer drug delivery systems, it is evident that non-bonded interaction energies such as vdW and electrostatic interactions between the enclosed or engrafted drugs and SWCNT determine the adsorption function of the drug on the surface or inside the SWCNT. It is however, important to gain a deeper understanding about the performance of SWCNTs as possible host nanocarriers of anticancer drugs through the calculation of interaction energies between the drug and the SWCNTs to ascertain their suitability as controlled drug delivery systems and gain an insight about their drug encapsulation and release functions.

The MD simulation in this regard, is a promising tool that has been widely applied to investigate the performance of complex nano-sized systems bridging diverse areas of cell biology and materials science^32–34^. Besides the great ability that the MD offers to evaluate of the thermal and mechanical features of nanostructures^35–40^, the method provides the ability to obtain a deeper insight into the interatomic interactions of nano-sized complex systems containing bio-molecules^41^. The anticancer drug Isatin (1H-indole-2,3-dione), a natural alkaloid in a form of red–orange powder (aka. Tribulin), derived from indole with a formula C_8_H_5_NO_2_ that was first obtained by Otto Linné Erdman and Auguste Laurent^42^ in 1840 as a by-product of indigo dye oxidation by nitric and chromic acids. Isatin is naturally found in a human body as an endogenous compound for metabolite of tryptophan or epinephrine^43^. Isatin is soluble in hot water, alcohol, acetic acid, and benzene but is sparingly soluble in ether. Remarkably, Isatin remains stable and soluble in concentrated hydrochloric acid and in concentrated sulfuric acid^44^ these properties allow encapsulation of Isatin as a guest molecule inside the host CNTs and theoretically permit an *in-situ* functionalization of Isatin-containing CNTs.

In the present work, first, the MD simulation was employed to evaluate the encapsulation of Isatin inside the end-capped SWCNT (10,10) host through the calculation of vdW between the host SWCNT and the drug, followed by computing the potential of mean force (PMF) of the encapsulated Isatin inside the SWCNT in a aqueous environment. Secondly, the storage capacity of the capped SWCNT (10,10) with the length of 30 Å to encapsulate the Isatin was evaluated.

## Simulation method

**Figure 1:**
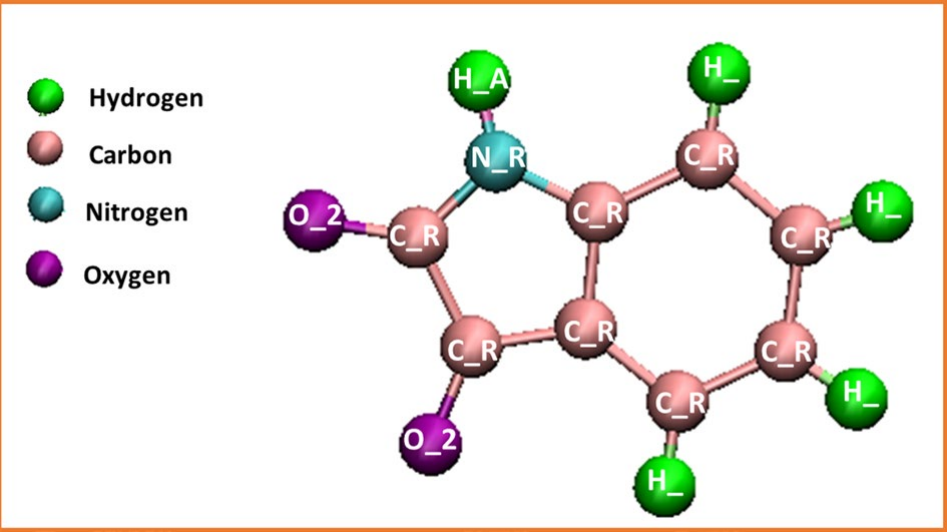
The molecular structure of anticancer drug Isatin and its atoms as represented using the DREIDING force field.

In the current work, the MD simulation of the encapsulation processes and storage of Isatin guest inside the capped SWCNT was performed by employing the Large-Scale Atomic/Molecular Simulator (LAMMPS) software (Ver. March 2018, https://www.lammps.org/download.html)^45^. Inspired by previous investigations, CHARMM27 force field was applied to obtain the interaction parameters of hydrogen and oxygen atoms in water molecules (TIP3P)^46^, a well-known force field widely applied in drug delivery simulation^47^. The Tersoff potential was used to consider the interaction between the carbon atoms in the SWCNT^48^. Evidently, several works have addressed successful application of the aforementioned force fields^49,50^. Atoms of modeled nanotube were fixed during the simulation. The molecular structure of Isatin is shown in Fig. 1. The interaction parameters between the atoms of Isatin were obtained from the DREIDING force filed, which is a smart approach successfully used in prediction of the structure and dynamics of organic, biological, and main-group inorganic molecules^51^. The optimization of geometry of the drug was carried out by means of CVff force field to find charges of atoms, as proposed by developers of DREIDING methodology^51^. The Isatin force field data can be found in supplementary data.

The temperature and the pressure of the systems were locked to 300 K and 101.3 kPa, respectively, by applying NPT Ensemble^52^. The cutoff distance for the Lennard–Jones potential and Coulombic potential was considered to be 12 Å. The parameters of Lennard–Jones potential for cross vdW interactions between non-bonded atoms was estimated using Lorentz–Berthelot combination rule^53^. The visualization was obtained by means of visual MD (VMD) simulation (Ver. 1.9.3, https://www.ks.uiuc.edu/Development/Download/download.cgi?PackageName=VMD)^54^.

The simulation steps of this research are described as following:

1. At first step, the insertion process of the peptide Isatin into the capped CNT, and subsequently the stability of encapsulated drug inside the capped SWCNT host were studied. Considering the size of the Isatin guest molecule, the capped SWCNT in order to serve as a host drug nano-carrier was selected the chirality of an armchair (10,10) and displaying the length of 30 Å. At the beginning of the MD simulation, Isatin was situated at the initial distance of 2 Å from the nanotube. The axial direction of the SWCNT was set parallel to the z-axis of the simulation box. The complex comprised of the capped SWCNT- Isatin was immersed in the simulation box consisting of TIP3P 3-point water molecules as well as counter-ions to neutralize the simulated solution with periodic boundary condition. To assess the encapsulation process of the peptide, in the first stage, the minimization of the system was performed in the canonical NVT ensemble at 300 K, where moles (N), volume (V) and temperature (T) gradients were conserved. Next, the MD runs were performed in the NPT ensemble for 15 ns with the time step of 1 fs.
  To evaluate the stability of the encapsulated Isatin inside the capped SWCNT, an external force was loaded on the encapsulated Isatin along the z-axis of the nanotube to pull it out from the capped CNT host in the direction opposite to the penetrating process. The spring constant *k* and pulling velocity were chosen equal to 15 kcal mol^−1^ Å^−2^ and 0.0005 Å ps^−1^, respectively^47^. The pulling process was simulated ten times to compute the potential of PMF using Jarzynski^'^s equality as noted below^46^:
  1 $$e^{ - \beta \Delta G} = e^{ - \beta W}$$
  where ΔG and W correspond to the free energy discrepancy between two states and the performed work on the system respectively. The *β* is equal to ($K_{B}$ T)^−1^, where $K_{B}$ stands for the Boltzmann constant.
2. At the second step, the storage capacity of the capped SWCNT (10,10) host was investigated. For this, 15 molecules of Isatin were placed inside the capped SWCNT. The axial directions of nanotubes were set to be parallel to the z-axis of the simulation box. The minimization of the system was done in the canonical NVT ensemble at 300 K while the SWCNT were fixed. Then, the MD run was performed in the NPT ensemble for 15 ns with the time step of 1 fs.

## Results and discussion

### Localization of Isatin within the capped SWCNT-drug complex

**Figure 2:**
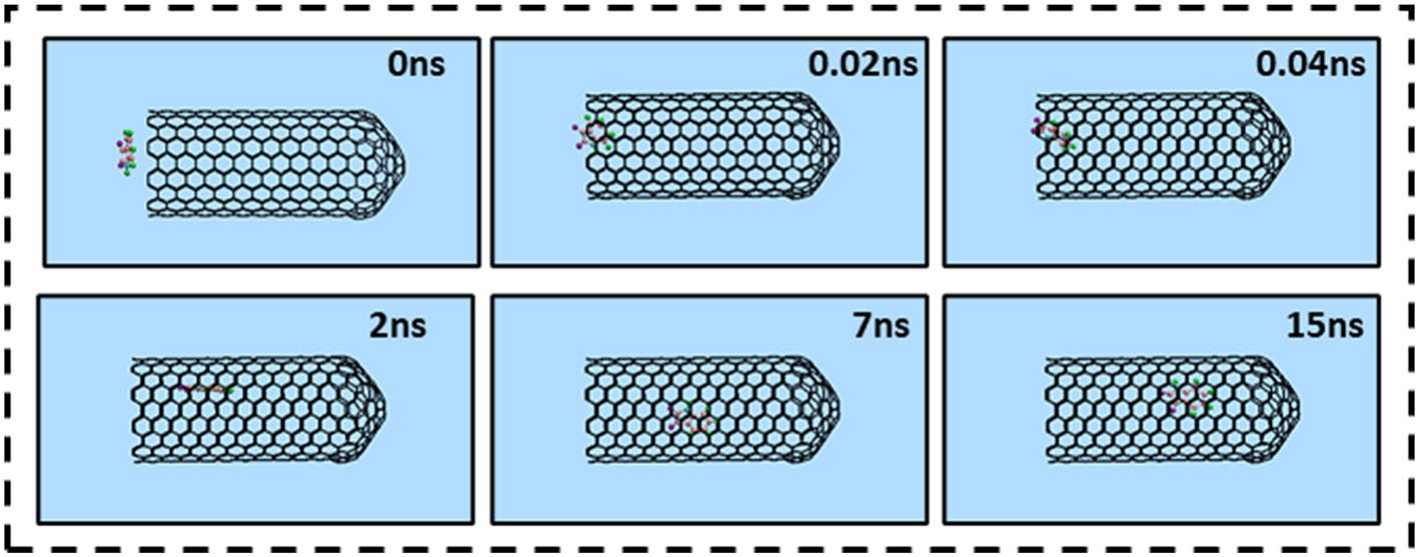
Representative snapshots of insertion of a single molecule of Isatin into an armchair capped SWCNT (10,10) host at various times. For clarity, the water molecules have been omitted from the image.

The encapsulation process of a single Isatin guest molecule inside the capped SWCNT (10,10) host was investigated by means of the MD simulation. Figure 2 illustrates the snapshots of positions of the drug at various times in the simulation box which obtained using VMD software. As shown in Fig. 2, Isatin molecule was successfully encapsulated inside the capped SWCNT host. Moreover, the drug remained stable in the cavity of the nanotube owing to the vdW interaction interactions between the Isatin’s conjugated aromatic rings and the interior wall (side wall and capped end) of the nanotube up to the end of the simulation time (15 ns). Another point is that DREIDNG, in the current work, appeared as a powerful potential for keeping the Istain's structure stable without any deformation. Therefore, it can be concluded that choosing this potential for the modeled anticancer drug was a smart choice^55^.

**Figure 3:**
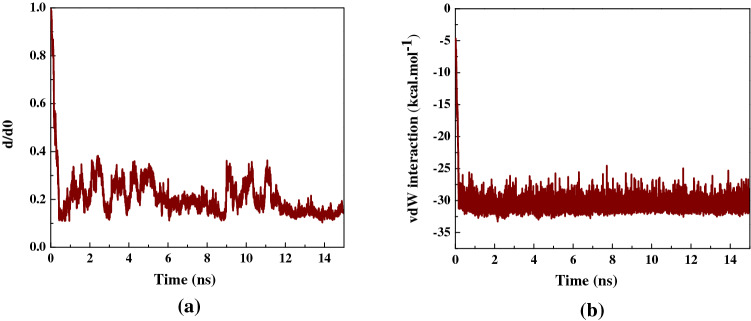
(**a**) The *d/d*_*0*_ values (normalized to the CoM distance, where *d*_*0*_ is the initial CoM distance) between the drug Isatin and capped SWCNT (10,10) as a function of simulation time, (**b**) the vdW interaction between the drug Isatin and the capped SWCNT as a function of simulation time.

Figure 3a shows the changes of the normalized center of mass (CoM) distance between Isatin guest and the capped SWCNT (10,10) host, *d*/*d*_0_, during the simulation. The *d*/*d*_0_ value was found to reduce significantly during the first 0.5 ns of the simulation in good agreement with rapid adsorption of the drug into the capped SWCNT cavity owing to the marked vdW interaction forces between Isatin and the nanotube. The same decreasing trend in *d/d*_*0*_ value of at the beginning of the encapsulation process was observed by Kang et al*.*^47^ reporting on SmtA protein experiencing self-adjustment through the conformational variations before entering the SWCNT. After the complete insertion of the drug occurred towards the end of the simulation, the value of the *d*/*d*_0_ were found to fluctuate continuously in the specified range due to the surrounding vdW interaction forces between the SWCNT host interior wall and the Isatin molecule.

According to the neutralized nature of the capped SWCNT (10,10), the value of the electrostatic interactions such as dipole–dipole, and H- bonding interactions between the nanotube and the Isatin molecule was found to be zero. Therefore, the vdW interaction energy was considered as an absorbance interaction between Isatin and the capped nanotube. Figure 3b demonstrates the changes in the value of the vdW interaction energy between the drug and the capped SWCNT (10,10) during the simulation.

Evidently, once the simulation was initialized, the value of the vdW energy decreased significantly from − 5 kcal mol^−1^ to approximately − 30 kcal mol^−1^ and fluctuated around the latter value until the end of the simulation. These changes revealed that the Isatin-SWCNT vdW interaction energy that is attributed to the relatively small size of the drug molecule, caused a rapid adsorption of Isatin molecule into the capped SWCNT host volume cavity. Moreover, the obtained low value for the vdW energy shows that a desirable interaction between the guest molecule and SWCNT host occurred in favor of both the adsorption and desorption functions of the drug in the Isatin-SWCNT nano-delivery system^56^.

After the sudden drop, the smooth fluctuation of the value of the vdW energy around the specified value showed that Isatin was affected by the vdW interaction between its conjugated aromatic rings of the and the interior wall (side wall and capped end) of the nanotube and remained stably encapsulated until to the end of the simulation. A similar variation in vdW values was reported by Maleki et al*.*^26^ determining the electrostatic and vdW interactions between the Doxorubicin molecule and SWCNT. According to their results, a zero value of electrostatic interactions provides the absorbance of Doxorubicin on the SWCNT rendered by the vdW interactions with a favorable negative value. An MD simulation performed by Hashemzadeh et al.^57^ revealed that a reduction of the vdW energy between the guest Paclitaxel (AKA. Taxol) and SWCNT host to the negative value, which supports the drug natural encapsulation inside the SWCNT.

### Calculation of free energy from the MD simulation

**Figure 4:**
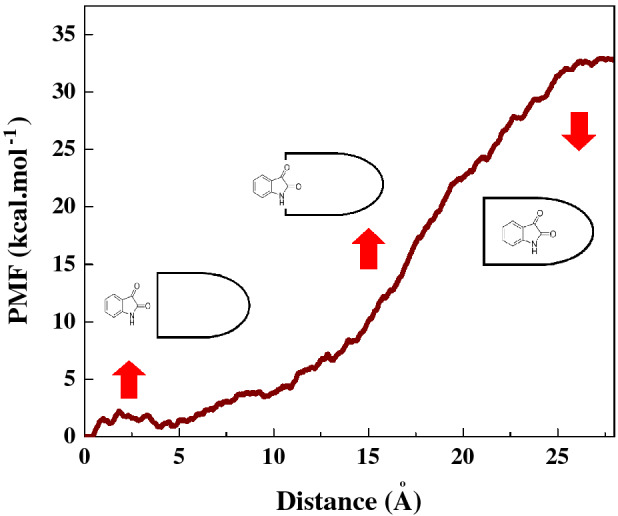
The PMF for the system comprised of a single Isatin guest molecule inside the capped SWCNT host computed from ten (10) pulling through the MD simulation**.** The images represent the positions of Isatin molecule corresponding to the z-coordinate along the capped SWCNT at a close proximity-, entering/exiting- and in a fully-enclosed position inside the SWCNT host.

Following the termination of the MD simulation, once Isatin was completely inserted inside the capped SWCNT (10,10) at 15 ns, the PMF profile of the encapsulated drug was determined through pulling the drug molecule out by means of the MD simulation at the speed of 0.0005 Å ps^−1^, which has been selected according to the encapsulation process speed. Repeating the simulation ten times the average value of work (W) at each pulling distance, was obtained as shown in the PMF functional profile (see Fig. 4) that illustrates the Isatin’s positions along the z-axis of the capped SWCNT host relative to the distance travelled in Fig. 4. The free energy of this simulated system was found to increase relative to the pulling distance reaching its maximum value of 34 kcal mol^−1^ at the pulling distance of 28 Å. The free energy of encapsulated Isatin molecule is − 34 kcal mol^−1^, a signature of spontaneous encapsulation process. A similar value is reported by Veclani et al*.*^58^ for the Ciprofloxacin (AKA. Cipro, Cifran) antibiotic encapsulated in SWCNT as − 21 kcal mol^−1^, and − 9.5 kcal mol^−1^ for the drug adsorbed on the surface of a SWCNT.

### The variations in the conformation of Isatin

**Figure 5:**
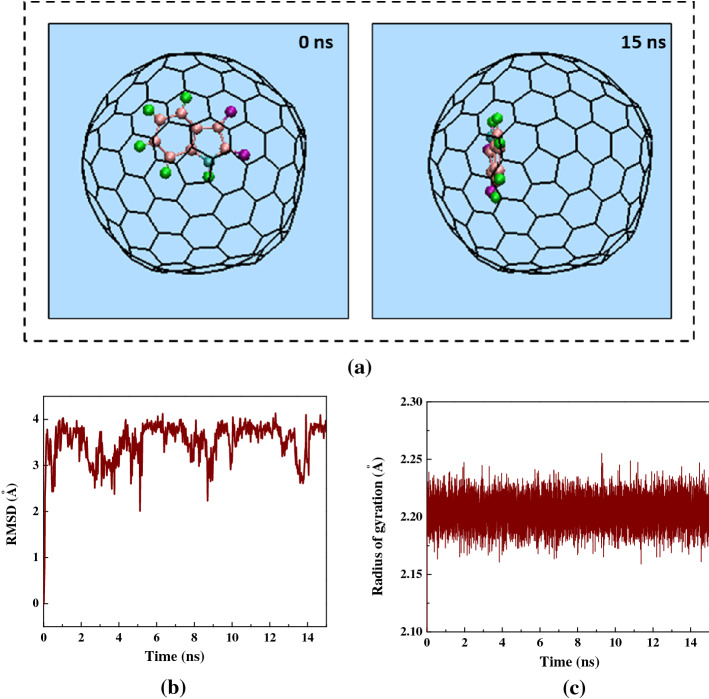
(**a**) Axial views of a single Isatin guest molecule at 0 ns and 15 ns in the MD simulation. For clarity, the water molecules have been omitted from the image. (**b**) The RMSD values of Isatin as shown as a function of simulation time. (**c**) Isatin radius gyration is shown as a function of simulation time in the capped SWCNT- Isatin complex.

The alterations in the conformation of a single Isatin molecule at 0 and 15 ns of the MD simulation are shown in Fig. 5a. The left image in Fig. 5a depicts the drug molecule immersed in aqueous solution outside the capped SWCNT host at 0 ns. The right image in Fig. 5a corresponds to the state in which the conformation of the drug was adjusted to the interior wall of the SWCNT at 15 ns. Notably, at the end of the simulation, Isatin’s cyclic rings were adjusted to be situated parallel to the side wall of the SWCNT so that the highest the *π–π′* stacking interaction was established.

The root-mean-square-deviation (RMSD) corresponding to the variations in the conformation of Isatin inside the capped SWCNT (10,10) host as a function of the simulation time is shown in Fig. 5b. The RMSD varied significantly by 0.1 ns to the value of 4 Å which revealed the variations in the both the conformation and the position of the drug molecule under the influence of the capped SWCNT-Isatin vdW interaction. Following the insertion of Isatin molecule inside the capped SWCNT host, the value of RMSD was found to fluctuate in the specified range confirming that the conformation of the drug could not vary much due to the physical confinement inside the cavity of the capped SWCNT host. The similar changes in the RMSD value of the of platinum-based anticancer during the encapsulation inside the silicon-carbon nanotube was recently observed by Hasanzade et al*.*^59^. The alteration of the gyration radius of Isatin is shown in Fig. 5c. Isatin gyration radius was found to be essentially unaltered but varied continuously in a narrow range of 2.175–2.225 Å owing to a relatively small size and rigid molecular structure of the drug.

**Figure 6:**
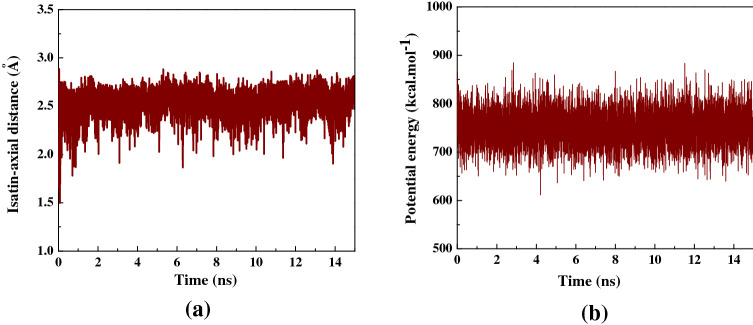
(**a**) The distance between Isatin’s CoM and the central axis of capped SWCNT as a function of simulation time. (**b**) The potential energy variation of Isatin inside the capped SWCNT-Isatin complex as a function of simulation time.

Figure 6a shows the alterations in distance between Isatin’s CoM and the central axis of the capped SWCNT (10,10) host as a function of simulation time. At the beginning of the simulation, the drug in the simulation box was positioned in a way that the CoM of the guest drug was aligned on the central axis of the SWCNT host. Once the simulated commenced, Isatin molecule moved suddenly towards one side of the capped SWCNT owing to vdW interaction between the SWCNT wall and the drug resulting in an immediate increase of the drug-axis distance to the value of 2.7 Å. After the complete insertion of Isatin molecule inside the SWCNT host, the distance between the CoM of the drug and central axis of the SWCNT host fluctuated slightly confirming the stability of Isatin molecule inside the SWCNT due to the desirable SWCNT-to-Isatin vdW interaction.

The changes in the potential energy of Isatin during the self-encapsulation process is shown Fig. 6b. The potential energy curve of Isatin is essentially a linear function that shows minor fluctuations around the value of 600 kcal mol^−1^ during the insertion process confirming that the simulated system has reached its equilibrium state. A similar trend in potential energy changes of the Flutamide (AKA. Eulexin) anticancer drug in the SWCNT- Flutamide complex was recently reported by Kamel et al.^60^.

### The storage of Isatin inside the capped SWCNT

**Figure 7:**
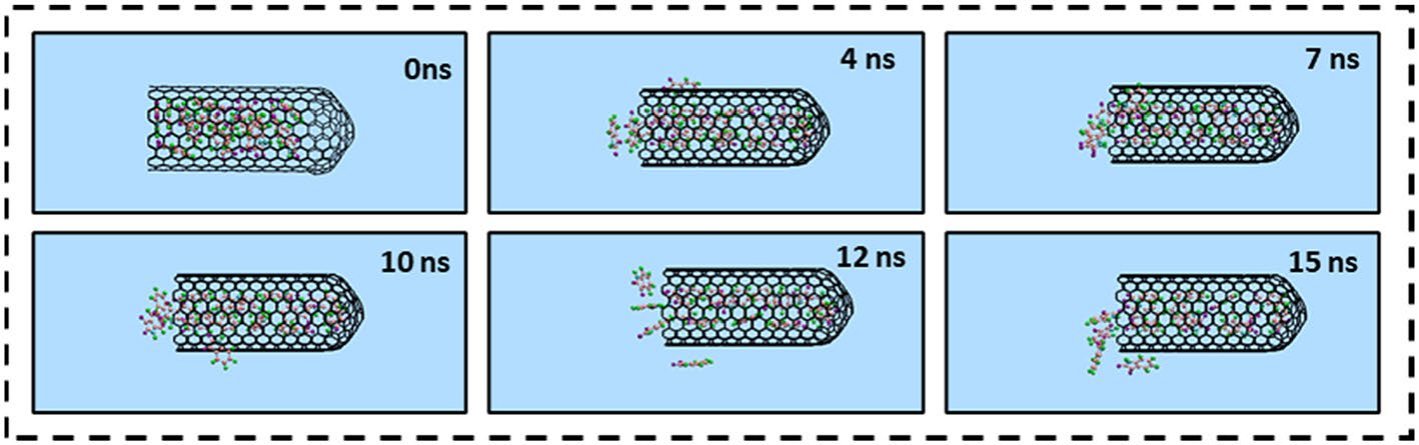
Representative snapshots of storage capacity of Isatin guest molecules inside a single capped SWCNT (10,10) host at various simulation times. For clarity, the water molecules have been omitted from the image.

Due to the possible cytotoxicity associated with CNTs at high concentrations, the encapsulation of more than one guest Isatin molecule inside a single host SWCNT is likely lower the amount the required CNTs in nanomedicine therapy. Figure 7 illustrates the snapshots of the MD simulation of the storage of the several Isatin guest molecules inside a single capped SWCNT host at various times.

**Figure 8:**
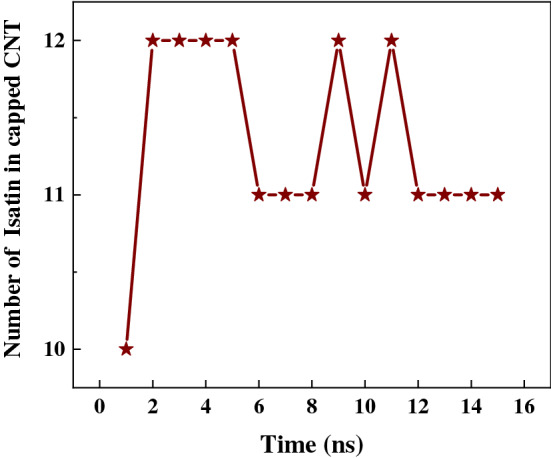
A potential number of Isatin molecules enclosed inside a single capped SWCNT (10,10) host carrier as a function of simulation time.

Figure 8 shows the number of Isatin guest molecules encapsulated in a single capped SWCNT (10,10) host as a function of simulation time. At 0 ns of the simulation, fifteen (15) molecules of Isatin were positioned inside the cavity of a single capped SWCNT host. As the simulation commenced, a few Isatin molecules were expelled from the SWCNT cavity and shown attached to the surrounding exterior wall of the SWCNT due to the vdW interactions. Out simulation have shown that an average eleven (11) Isatin guest molecules could be stably encapsulated inside a single capped SWCNT host carrier.

## Conclusion

Our work described the encapsulation process of the anticancer drug Isatin inside the capped SWCNT (10,10) and, evaluated a number of Isatin guest molecules that can be readily encapsulated inside the SWCNT cavity employing the MD simulation. Our results revealed that a single Isatin guest molecule can be readily and rapidly adsorbed inside the capped SWCNT host and remained stably encapsulated until the end of the MD simulation. The value of the capped SWCNT-Isatin vdW interaction energy was found to reach the value of − 30 kcal mol^−1^ at the end of the simulation (15 ns) that showed a high probability of complete adsorption and desorption of Isatin guest inside the SWCNT host SWCNT-Isatin drug nano-delivery system. In first 0.1 ns of the simulation, the RMSD was found to vary significantly reaching the value of 4 Å. The latter revealing the variations in both the conformation and the position of Isatin guest under the influence of the capped SWCNT-Isatin vdW interaction. The obtained value of free energy of encapsulation process, namely, − 34 kcal mol^−1^ has confirmed that the insertion procedure of Isatin guest into the SWCNT host occurred spontaneously and naturally. The MD simulation performed to calculate the probable storage capacity of the capped SWCNT (10,10) with length of 30 Å have shown that approximately eleven (11) Isatin guest molecules could be stably encapsulated inside a single capped SWCNT host cavity, confirming a potential for SWCNT-Isatin drug delivery systems in future nano-medicine applications.

## Supplementary Information

Supplementary Information.

## References

[1] Edis Z, Wang J, Waqas MK, Ijaz M, Ijaz M (2021). Nanocarriers-mediated drug delivery systems for anticancer agents: An overview and perspectives. Int. J. Nanomed..

[2] Jin K-T (2020). Recent trends in nanocarrier-based targeted chemotherapy: Selective delivery of anticancer drugs for effective lung, colon, cervical, and breast cancer treatment. J. Nanomater..

[3] Pucci C, Martinelli C, Ciofani G (2019). Innovative approaches for cancer treatment: Current perspectives and new challenges. Ecancermed. Sci..

[4] Zhou Q, Zhang L, Yang T, Wu H (2018). Stimuli-responsive polymeric micelles for drug delivery and cancer therapy. Int. J. Nanomed..

[5] Edis Z, Wang J, Waqas MK, Ijaz M, Ijaz M (2021). Nanocarriers-mediated drug delivery systems for anticancer agents: An overview and perspectives. Int. J. Nanomed..

[6] Smith AW (2005). Biofilms and antibiotic therapy: Is there a role for combating bacterial resistance by the use of novel drug delivery systems?. Adv. Drug Deliv. Rev..

[7] Zarrintaj, P. *et al.* Poloxamer: A versatile tri-block copolymer for biomedical applications. **110**, 37–67 (2020). 10.1016/j.actbio.2020.04.028 32417265

[8] Yoosefian M, Etminan N (2016). Density functional theory (DFT) study of a new novel bionanosensor hybrid; tryptophan/Pd doped single walled carbon nanotube. Physica E.

[9] Skandani AA, Al-Haik M (2013). Reciprocal effects of the chirality and the surface functionalization on the drug delivery permissibility of carbon nanotubes. Soft Matter.

[10] Gu FX (2007). Targeted nanoparticles for cancer therapy. Nano Today.

[11] Shaki H, Raissi H, Mollania F, Hashemzadeh H (2019). Modeling the interaction between anti-cancer drug penicillamine and pristine and functionalized carbon nanotubes for medical applications: Density functional theory investigation and a molecular dynamics simulation. J. Biomol. Struct. Dyn..

[12] Ramos MADS (2018). Nanotechnology-based drug delivery systems for control of microbial biofilms: A review. Int. J. Nanomed..

[13] Bernkop-Schnürch A, Bratengeyer I, Valenta C (1997). Development and in vitro evaluation of a drug delivery system protecting from trypsinic degradation. Int. J. Pharm..

[14] Dehaghani MZ (2020). Insight into the self-insertion of a protein inside the boron nitride nanotube. ACS Omega.

[15] Peer D (2007). Nanocarriers as an emerging platform for cancer therapy. Nat. Nanotechnol..

[16] Dehaghani MZ (2021). Boron nitride nanotube as an antimicrobial peptide carrier: A theoretical insight. Int. J. Nanomed..

[17] Arsawang U (2011). How do carbon nanotubes serve as carriers for gemcitabine transport in a drug delivery system?. J. Mol. Graph. Model..

[18] Yazdi, M. K., Saeidi, H., Zarrintaj, P., Saeb, M. R. & Mozafari, M. In *Fundamentals and Emerging Applications of Polyaniline* 143–163 (Elsevier, 2019).

[19] Jouyandeh M (2020). Highly curable self-healing vitrimer-like cellulose-modified halloysite nanotube/epoxy nanocomposite coatings. Chem. Eng. J..

[20] Elhissi AMA, Ahmed W, Hassan IU, Dhanak VR, D'Emanuele A (2012). Carbon nanotubes in cancer therapy and drug delivery. J. Drug Deliv..

[21] Imani Yengejeh, S., Rybachuk, M., Kazemi, S. A. & Öchsner, A. In *Encyclopedia of Continuum Mechanics* (eds H. Altenbach & A. Öchsner) 1–14 (Springer, Berlin, 2018).

[22] Yengejeh SI, Öchsner A, Kazemi SA, Rybachuk M (2018). Numerical analysis of the structural stability of ideal (defect-free) and structurally and morphologically degenerated homogeneous, linearly- and angle-adjoined nanotubes and cylindrical fullerenes under axial loading using finite element method. Int. J. Appl. Mech..

[23] Ghavamian, A., Rybachuk, M. & Öchsner, A. in *Defects in Advanced Electronic Materials and Novel Low Dimensional Structures* (eds J. Stehr, I. Buyanova, & W. Chen) 87–136 (Woodhead Publishing, 2018).

[24] Moradnia H, Raissi H, Shahabi M (2020). The performance of the single-walled carbon nanotube covalently modified with polyethylene glycol to delivery of Gemcitabine anticancer drug in the aqueous environment. J. Biomol. Struct. Dyn..

[25] Roosta S, Hashemianzadeh SM, Ketabi S (2016). Encapsulation of cisplatin as an anti-cancer drug into boron-nitride and carbon nanotubes: Molecular simulation and free energy calculation. Mater. Sci. Eng. C.

[26] Maleki R, Afrouzi HH, Hosseini M, Toghraie D, Rostami S (2020). Molecular dynamics simulation of Doxorubicin loading with N-isopropyl acrylamide carbon nanotube in a drug delivery system. Comput. Methods Prog. Biomed..

[27] Mirsalari H, Maleki A, Raissi H, Soltanabadi A (2021). Investigation of the pristine and functionalized carbon nanotubes as a delivery system for the anticancer drug dacarbazine: Drug encapsulation. J. Pharm. Sci..

[28] Dehneshin N, Raissi H, Hasanzade Z, Farzad F (2019). Using molecular dynamics simulation to explore the binding of the three potent anticancer drugs sorafenib, streptozotocin, and sunitinib to functionalized carbon nanotubes. J. Mol. Model..

[29] Panczyk T, Wolski P, Lajtar L (2016). Coadsorption of doxorubicin and selected dyes on carbon nanotubes. Theoretical investigation of potential application as a pH-controlled drug delivery system. Langmuir.

[30] Panczyk T, Jagusiak A, Pastorin G, Ang WH, Narkiewicz-Michalek J (2013). Molecular dynamics study of cisplatin release from carbon nanotubes capped by magnetic nanoparticles. J. Phys. Chem. C.

[31] Wolski P, Narkiewicz-Michalek J, Panczyk M, Pastorin G, Panczyk T (2017). Molecular dynamics modeling of the encapsulation and de-encapsulation of the carmustine anticancer drug in the inner volume of a carbon nanotube. J. Phys. Chem. C.

[32] Katiyar RS, Jha PK (2018). Molecular simulations in drug delivery: Opportunities and challenges. Wiley Interdiscip. Rev. Comput. Mol. Sci..

[33] Singh A, Vanga SK, Orsat V, Raghavan V (2018). Application of molecular dynamic simulation to study food proteins: A review. Crit. Rev. Food Sci. Nutr..

[34] Zarghami Dehaghani M (2021). Boron nitride nanotube as an antimicrobial peptide carrier: A theoretical insight. Int. J. Nanomed..

[35] Bagheri B (2020). Correlation between surface topological defects and fracture mechanism of γ-graphyne-like boron nitride nanosheets. Comput. Mater. Sci..

[36] Dehaghani MZ (2020). Fracture toughness and crack propagation behavior of nanoscale beryllium oxide graphene-like structures: A molecular dynamics simulation analysis. Eng. Fract. Mech..

[37] Dehaghani MZ (2021). Fracture mechanics of polycrystalline beryllium oxide nanosheets: A theoretical basis. Eng. Fract. Mech..

[38] Bagheri B (2021). Fracture fingerprint of polycrystalline C3N nanosheets: Theoretical basis. J. Mol. Graph. Model..

[39] Salmankhani A (2021). A theoretical scenario for the mechanical failure of boron carbide nanotubes. Comput. Mater. Sci..

[40] Albooyeh A, Dadrasi A, Mashhadzadeh AH (2020). Effect of point defects and low-density carbon-doped on mechanical properties of BNNTs: A molecular dynamics study. Mater. Chem. Phys..

[41] Fatemi SM, Foroutan M (2017). Review of recent studies on interactions between polymers and nanotubes using molecular dynamic simulation. J. Iran. Chem. Soc..

[42] Laurent A (1840). Recherches sur l’indigo. Ann. Chim. Phys.

[43] de Paiva, R. E. F., Vieira, E. G., da Silva, D. R., Wegermann, C. A. & Ferreira, A. M. C. Anticancer compounds based on Isatin-derivatives: Strategies to ameliorate selectivity and efficiency. *Front. Mol. Biosci.* **7**, 1–24 (2020). 10.3389/fmolb.2020.627272 PMC7889591 33614708

[44] Sumpter WC (1944). The chemistry of Isatin. Chem. Rev..

[45] Plimpton S (1995). Fast parallel algorithms for short-range molecular dynamics. J. Comput. Phys..

[46] Park S, Schulten K (2004). Calculating potentials of mean force from steered molecular dynamics simulations. J. Chem. Phys..

[47] Kang Y (2009). On the spontaneous encapsulation of proteins in carbon nanotubes. Biomaterials.

[48] Los J (2017). Extended Tersoff potential for boron nitride: Energetics and elastic properties of pristine and defective h-BN. Phys. Rev. B.

[49] Rukmani SJ, Kupgan G, Anstine DM, Colina CM (2019). A molecular dynamics study of water-soluble polymers: Analysis of force fields from atomistic simulations. Mol. Simul..

[50] Nejad MA, Urbassek HM (2019). Diffusion of cisplatin molecules in silica nanopores: Molecular dynamics study of a targeted drug delivery system. J. Mol. Graph. Model..

[51] Mayo SL, Olafson BD, Goddard WA (1990). DREIDING: A generic force field for molecular simulations. J. Phys. Chem..

[52] Feller SE, Zhang Y, Pastor RW, Brooks BR (1995). Constant pressure molecular dynamics simulation: The Langevin piston method. J. Chem. Phys..

[53] Hirschfelder JO, Curtiss CF, Bird RB, Mayer MG (1964). Molecular Theory of Gases and Liquids.

[54] Humphrey W, Dalke A, Schulten K (1996). VMD: Visual molecular dynamics. J. Mol. Graph..

[55] Karimipour A (2021). Molecular dynamics performance for coronavirus simulation by C, N, O, and S atoms implementation dreiding force field: Drug delivery atomic interaction in contact with metallic Fe, Al, and steel. Comput. Part. Mech..

[56] Hasanzade Z, Raissi H (2020). Carbon and boron nanotubes as a template material for adsorption of 6-Thioguanine chemotherapeutic: A molecular dynamics and density functional approach. J. Biomol. Struct. Dyn..

[57] Hashemzadeh H, Raissi H (2017). The functionalization of carbon nanotubes to enhance the efficacy of the anticancer drug paclitaxel: A molecular dynamics simulation study. J. Mol. Model..

[58] Veclani D, Melchior A (2020). Adsorption of ciprofloxacin on carbon nanotubes: Insights from molecular dynamics simulations. J. Mol. Liquids.

[59] Khatti Z, Hashemianzadeh SM, Shafiei SA (2018). A molecular study on drug delivery system based on carbon nanotube compared to silicon carbide nanotube for encapsulation of platinum-based anticancer drug. Adv. Pharm. Bull..

[60] Kamel M, Raissi H, Morsali A (2017). Theoretical study of solvent and co-solvent effects on the interaction of Flutamide anticancer drug with Carbon nanotube as a drug delivery system. J. Mol. Liq..

