# Supplementary material for: Encapsulation of an anticancer drug Isatin inside a host nano-vehicle SWCNT: a molecular dynamics simulation
Source: Sci Rep. 2021 Sep 21;11:18753. doi: 10.1038/s41598-021-98222-2 (PMC8455564; doi:10.1038/s41598-021-98222-2)
Supplement: Supplementary file 1 — Supplementary Information. [file 41598_2021_98222_MOESM1_ESM.pdf]

Supplementary information

Encapsulation of an anticancer drug Isatin inside a capped SWCNT: a molecular dynamics simulation

Maryam Zarghami Dehaghani[1], Farrokh Yousefi[2], Farzad Seidi[1], Babak Bagheri[3], Amin Hamed Mashhadzadeh[4,*], Ghasem Naderi[5], Amin Esmaeili[6], Otman Abida[7], Sajjad Habibzadeh[8,*], Mohammad Reza Saeb[9], Maksym Rybachuk[10,11,12]

[1] Jiangsu Co-Innovation Center of Efficient Processing and Utilization of Forest Resources and Joint International Research Lab of Lignocellulosic Functional Materials, Nanjing Forestry University, Nanjing 210037, China

[2] Department of Physics, University of Zanjan, 45195-313, Zanjan, Iran

[3] Department of Chemical and Biomolecular Engineering, Korea Advanced Institute of Science and Technology (KAIST), Daejeon 305-701, Republic of Korea

[4] Mechanical and Aerospace Engineering, School of Engineering and Digital Sciences, Nazarbayev University, Nur-Sultan 010000, Kazakhstan

[5] Iran Polymer and Petrochemical Institute (IPPI), Tehran, Iran

[6] Department of Chemical Engineering, College of the North Atlantic—Qatar, 24449 Arab League St, P.O. Box 24449 Doha, Qatar

[7] College of Engineering and Technology, American University of the Middle East, Kuwait

[8] Department of Chemical Engineering, Amirkabir University of Technology (Tehran Polytechnic), Tehran, Iran

[9] Center of Excellence in Electrochemistry, School of Chemistry, College of Science, University of Tehran, Iran

[10] School of Engineering and Built Environment, Griffith University, 170 Kessels Rd, Nathan QLD 4111, AUSTRALIA

[11] Queensland Micro- and Nanotechnology Centre, Griffith University, West Creek Road, Nathan QLD 4111, AUSTRALIA

[12] Centre for Quantum Dynamics and Australian Attosecond Science Facility, Griffith University, Science Road, Nathan QLD 4111, AUSTRALIA

The potential energy of the molecule Isatin (E) is defined as below:

$$E = E_{val} + E_{nb} \quad (1)$$

Where E_{val} is valence or bonded interactions, and E_{nb} refers to non-bonded interactions which are the superposition of the van der Waals energy (E_{vdW}) and Coulombic energy (as shown in Eq. 2)

$$E_{nb} = E_{vdW} + E_Q \quad (2)$$

E_{vdW} depends on the two parameters of D_0 and R_0 , which are van der Waals well depth (kcal/mol), and van der Waals bond length (as defined in Eq. 3). The obtained values of the D_0 and R_0 for each atom type of the drug Isatin are tabulated in Table. 1.

$$E_{vdW} = D_0((\frac{R}{R_0})^{-12} - 2(\frac{R}{R_0})^{-6}) \quad (3)$$

Table 1. Van der Waals parameters of Isatin atoms

Atom Type	R_0 (Å)	D_0 (Kcal.mol^{-1})
C_R	3.880	0.0950
N_R	3.695	0.1450
O_2	3.510	0.2150
H_	3.200	0.0100
H___A	3.200	0.0100

The valence interaction comprises of the bonding stretch (E_B), bond-angle bend (E_A), dihedral angle torsion (E_T), and inversion terms (E_I) as represented in Eq. 4:

$$E_{val} = E_B + E_A + E_T + E_I \tag{4}$$

The Bond stretch interaction is described as below:

$$E_B = K_e(R - R_e)^2 \tag{5}$$

The force constant (K_e), and anharmonic terms near equilibrium (R_e) for bond Types of the drug Isatin are reported in Table 2.

Table 2. K_e and R_e constants for bond types of drug Isatin

Bond Type	K_e (Kcal.mol^{-1}. Å^{-2})	R_e (Å)
C_R-C_R	525	1.39
C_R-H_	350	1.02
C_R-N_R	350	1.34
C_R-O_2	700	1.25
N_R-H___A	350	0.97

The bond-angle bend interaction energy (E_A) between is defined in Eq. 6. The values of equilibrium angle (θ_J°) and force constant (K_{IJK}) for angle types of the drug Isatin are summarized in Table 3.

$$E_A = E_{IJK} = K_{IJK}(\theta_{IJK} - \theta_J^{\circ})^2 \qquad (6)$$

Table 3. θ_J° and K_{IJK} for angle types of the drug Isatin.

**Angle Type**	K_{IJK} **(kcal.mol^{-1}.rad^{-2})**	θ_J° **(rad)**
C_R-C_R-C_R	50	120.00
C_R-C_R-H_	50	120.00
C_R-C_R-N_R	50	120.00
C_R-C_R-O_2	50	120.00
N_R-C_R-O_2	50	120.00
C_R-N_R-C_R	50	120.00
C_R-N_R-H___A	50	120.00

The torsion or dihedral interaction energy (E_D) for two bonds IJ and KL connected with bond is defined as below:

$$E_D = E_{IJKL} = V_{JK}(1 - \cos(n_{IL}(\varphi - \varphi_{JK}^{\circ}))) \qquad (7)$$

Where V_{JK}, n_{IL}, and φ°_{JK} correspond to barrier to rotation, the periodicity, and the equilibrium angle constants. The values of these constants for dihedral types of the drug Isatin are summarized in Table 4.

Table 4. The values of V_{JK}, n_{IL}, and φ°_{JK} for dihedral types of the drug Isatin

**Dihedral Type**	V_{JK} **(Kcal.mol^{-1})**	n_{IL}	φ°_{JK}
C_R-C_R-C_R-C_R	5	2	0
C_R-C_R-C_R-H_	5	2	0
H_-C_R-C_R-H_	5	2	0
C_R-C_R-C_R-N_R	5	2	0
N_R-C_R-C_R-H_	5	2	0
C_R-C_R-N_R-C_R	5	2	0
C_R-C_R-N_R-H___A	5	2	0
C_R-C_R-C_R-O_2	5	2	0
N_R-C_R-C_R-O_2	5	2	0
O_2-C_R-C_R-O_2	5	2	0
O_2-C_R-N_R-C_R	5	2	0
O_2-C_R-N_R-H___A	5	2	0

The inversion (improper) interaction energy (E_I) for drug Isatin with planar equilibrium geometry is considered in Eq. 8.

$$E_I = E_{IJKL} = K_I(1 - cos\Psi_I) \qquad (8)$$

Where Ψ_I is and angle between the IL bond and JIK plane. The values of force constant K_I for improper types of the drug Isatin are listed in Table 5.

Table 5. values of K_I for improper types of the drug Isatin

**Improper Type**	**K_I (kcal.mol-^{1}.rad^{-2})**
C_R-C_R-C_R-H_	40.00
C_R-C_R-C_R-N_R	40.00
C_R-C_R-C_R-C_R	40.00
C_R-C_R-C_R-O_2	40.00
C_R-C_R-N_R-O_2	40.00
C_R-N_R-C_R-H___A	40.00
